# A Comprehensive Review on Pharmacological Activities of Pachypodol: A Bioactive Compound of an Aromatic Medicinal Plant Pogostemon Cablin Benth

**DOI:** 10.3390/molecules28083469

**Published:** 2023-04-14

**Authors:** Sehrish Fatima, Iqra Farzeen, Asma Ashraf, Bilal Aslam, Muhammad Umar Ijaz, Sumreen Hayat, Muhammad Hassan Sarfraz, Saima Zafar, Nimrah Zafar, Jeremiah Oshiomame Unuofin, Sogolo Lucky Lebelo, Saima Muzammil

**Affiliations:** 1 Department of Zoology, Government College University, Faisalabad 38000, Pakistan; sherishl83@gmail.com (S.F.); iqrafar@yahoo.com (I.F.); asmabinm@gmail.com (A.A.); zafarsaima@yahoo.com (S.Z.); nimrazaf@yahoo.com (N.Z.); 2 Institute of Microbiology, Government College University, Faisalabad 38000, Pakistan; baslam551@gmail.com (B.A.); sumreen.hayat84@gmail.com (S.H.); hassan2008@gmail.com (M.H.S.); 3 Department of Zoology, Wildlife and Fisheries, University of Agriculture, Faisalabad 38040, Pakistan; umar.ijaz@uaf.edu.pk; 4 Department of Life and Consumer Sciences, College of Agriculture and Environmental Sciences, Private Bag X06, Florida 1710, South Africa; unuofinjeremiah@gmail.com (J.O.U.);

**Keywords:** *Pogostemon cablin*, anti-microbial, anti-cancerous, anti-inflammatory, flavonoids, essential oil, pachypodol

## Abstract

As is well known, plant products have been increasingly utilized in the pharmaceutical industry in recent years. By combining conventional techniques and modern methodology, the future of phytomedicines appears promising. *Pogostemon Cablin* (patchouli) is an important herb used frequently in the fragrance industries and has various therapeutic benefits. Traditional medicine has long used the essential oil of patchouli (*P. cablin*) as a flavoring agent recognized by the FDA. This is a gold mine for battling pathogens in China and India. In recent years, this plant has seen a significant surge in use, and approximately 90% of the world’s patchouli oil is produced by Indonesia. In traditional therapies, it is used for the treatment of colds, fever, vomiting, headaches, and stomachaches. Patchouli oil is used in curing many diseases and in aromatherapy to treat depression and stress, soothe nerves, regulate appetite, and enhance sexual attraction. More than 140 substances, including alcohols, terpenoids, flavonoids, organic acids, phytosterols, lignins, aldehydes, alkaloids, and glycosides, have been identified in *P. cablin*. Pachypodol (C18H16O7) is an important bioactive compound found in *P. cablin.* Pachypodol (C18H16O7) and many other biologically essential chemicals have been separated from the leaves of *P. cablin* and many other medicinally significant plants using repeated column chromatography on silica gel. Pachypodol’s bioactive potential has been shown by a variety of assays and methodologies. It has been found to have a number of biological activities, including anti-inflammatory, antioxidant, anti-mutagenic, antimicrobial, antidepressant, anticancer, antiemetic, antiviral, and cytotoxic ones. The current study, which is based on the currently available scientific literature, intends to close the knowledge gap regarding the pharmacological effects of patchouli essential oil and pachypodol, a key bioactive molecule found in this plant.

## 1. Introduction

Medicinal plants make up a sizable portion of flora and a significant source of raw ingredients for the aroma, cosmetic, culinary, and fragrance industries. Despite significant advancements in the study of synthetic drugs, plants, and the products they produce are still widely used in the pharmaceutical business and are still regarded as the main sources of medicines [1,2]. The majority of current medications are made from plants and are obtained by combining modern technology with conventional methods [3]. In several indigenous and traditional medical systems in India, including Ayurveda, Unani, Sidda, and others, the use of plants is usual [4]. The herbs employed in traditional medicine are among these plants, and they are a gold mine for fighting pathogens. Recently, traditional Indian plants have attracted considerable attention. A member of the Lamiaceae family, *P. cablin* (patchouli) is a fragrant herb with significant commercial value [5].

Patchouli is a significant herb frequently used in the fragrance industry, and it has a number of therapeutic advantages. Patchouli oil is an FDA-approved flavoring ingredient that has been used in traditional medicine for a very long time to treat colds, fever, vomiting, headaches, and stomachaches. Several disorders are treated using patchouli oil, including aromatherapy to cure depression and stress and increase sexual attraction.

*P. cablin* contains different biologically active compounds, including alcohols, terpenoids, flavonoids, organic acids, phytosterols, lignins, aldehydes, alkaloids, and glycosides. These compounds possess many biological activities, such as anti-inflammatory, antioxidant, anti-mutagenic, antimicrobial, antidepressant, anti-cancer, anti-emetic, antiviral, and cytotoxic ones.

In India and China, the patchouli plant has long been utilized in conventional medicine to treat a number of illnesses. It has been applied in Chinese medicine as an anti-emetic, to combat moisture, to lessen summer heat and external syndromes, and to increase appetite [6]. Due to this fact, a sizable number of researchers have examined possible therapeutic candidates from patchouli [7]. Puripattanavong et al. (2015) [8] have given considerable attention to this plant. Inflammatory illnesses are treated with Chinese traditional formulas like Houdan Pill and Baoji Pill, which contain pachypodol [9,10].

The plant is a component of Indian Ayurvedic therapeutic formulations such as Virya, Rasa, and Guna. In Asian countries such as China, Malaysia, and Japan, it is used to treat bug bites, snake bites, stomachaches, headaches, nausea, and vomiting [6]. Globally, PC is being investigated to produce essential chemical components for developing new pharmacological compounds in the fragrance sector with superior clinical relevance. In addition to this, reports of flavonoids, lignins, alkaloids, and glycosides, among other phytochemical components, have been made. Sesquiterpene lactones are one of the main types of chemicals mentioned in PC [3].

The oil extracted from shade-dried patchouli leaves through steam distillation is what gives the plant its commercial significance. This herb’s essential oils can be extracted from a variety of parts, such as the leaves, flowers, stalks, branches, and roots [11]. Two conventional techniques for separating essential oils are hydro distillation (HD) and steam distillation (SD), both of which have gained universal support as methods of extraction [12]. The predominant fragrant, spicy scent of patchouli essential oil makes it highly prized in aromatherapy and perfumery. It functions as a solid base and offers long-lasting fixative qualities to stop evaporation and encourage tenacity [13].

It is frequently employed to produce various healthcare items in the fragrance industry,. This essential oil is widely used for aromatherapy to increase libido; treat melancholy, anxiety, and sleeplessness; soothe frazzled nerves; and relieve stress. Patchouli oil is also used for the treatment of skin conditions such as eczema, acne, and chapped and cracked skin [14]. The numerous therapeutic effects of patchouli essential oil include different effects such as ciratrisant, antithrombotic, fibrinolytic, febrifuge, anti-insecticidal, and antibacterial [15,16,17]. Essential oil of Patchouli has also been linked to a number of pharmacological effects, including sedative, anti-inflammatory, antiseptic, astringent, diuretic, and anti-mutagenic ones [18]. According to Wei and Shibamoto (2007) [19], menstrual cramps can be eased with a patchouli leaf infusion. Additionally, leaf essential oils are used to elevate mood and mental wellbeing. It is also included as a natural food flavoring component in the Food and Drug Administration’s (FDA) list of substances that are safe for consumption by human beings. Major food items including non-alcoholic and alcoholic drinks, gelatin meat, meat products, and frozen dairy dessert use the essential oil as a flavoring agent [20]. True patchouli (*P. cablin*) and java patchouli are two popular varieties of *P. heyneanus* (patchouli). It is a seed crop that is non-viable, and hybridization cannot produce variation in this plant. So far, there are not many well-established patchouli cultivars that can be grown for profit [21].

Patchouli can only be made variable through mutation breeding, which has been crucial in the last 50 years in the creation of excellent plant types with early maturity, high essential oil yield, and tolerance to both biotic and abiotic conditions [22,23]. Jor Lab P-1 (INGR18041) [23] is a new type of patchouli that was recently created by CSIR-NEIST (North-East Institute of Science and Technology, Jorhat, Assam, India, a constituent establishment of Council of Scientific and Industrial Research) using mutation breeding (gamma radiation). This variety is found to produce a lot of herbs and is able to manufacture essential oils. Indonesia, China, Malaysia, and Brazil are the world’s top patchouli essential oil producers. The projected annual global usage of patchouli essential oil is around 2000 tonnes [24].

The use of patchouli has Increased to roughly 300 tonnes annually, while production is less than 50 tonnes in India as a result of growth in the fields of areca nut and palm, tobacco, and other businesses, so India largely imports it, particularly from Indonesia. Indonesia produces approximately 90% of the world’s patchouli oil output, which is equal to 1000–1500 MT annually as stated in IFEAT2020 (International Federation of Essential Oils and Aroma Trades). However, because of a lack of high-yield or stress-tolerant cultivars and advanced agro-technologies, India trails far behind in terms of patchouli cultivation and essential oil extraction [25].

It has a potent sweet, herbaceous, fragrant, and spicy scent [26]. Because of its distinctive flavor and smell qualities, as well as its biological activity, patchouli is thought to have the greatest commercial potential of all the plants that produce essential oils (Bizzo et al., 2009). In combination with other ethereal oils, it creates a robust base, with a persistent quality and fixation that could stop evaporation, which helps to promote tenacity, even though it is not a main source of scent. This is why these natural oils are frequently utilized in the production of detergents, moisturizers, soaps, and fragrances [4]. In aromatherapy, the treatment of mood disorders, nervousness, and anxiety; hunger control; and heightened sexual attraction are all achieved with patchouli oil. Additionally, patchouli has antibacterial, antifungal, and insecticidal effects [27]. To create a thorough report on the biological features displayed by pure substances such as pachypodol, a compilation of the literature is made in this review. Utilizing a variety of search engines, including Google Scholar, Scopus, PubMed, and Science Direct, an updated research survey was conducted.

The review aims to emphasize the importance of *P. cablin* and pachypodol (a substance derived from its leaves) in therapeutics and pharmaceuticals. The previous research studies and reports on *P. cablin*’s properties and its significant biological activities are summarized in this literature along with suggestions for future research regarding the assessment of pachypodol as a potential substance showing numerous biological activities and medicinal benefits.

## 2. Botanical Description

Native to the Philippines, patchouli is now commercially grown in China, India, Singapore, Malaysia, Indonesia, Vietnam, and West Africa [28]. It also grows wild in several South Asian nations. In the Philippines, the native name of the patchouli plant is “cablin” and “cabalam”, both of which are related to each other [29]. There are reportedly different types of pogostemon in India. In addition to being known as patchouli, this plant is also referred to as patchapan/patcha in Sanskrit; tamala patra in Marathi; patchetene in Kannada; pacchilai in Tamil; guang hou xiang in China; patchilla in Malayalam; nilam in Malaysia, and phimsen in Thailand [18].

Patchouli is a tough perennial herb with morphological adaptations to hot and muggy climates. It has tall stems and large leaves, and it can reach heights of 1 to 1.2 m (0.85 inches). The dorsal surface of the leaves has an abundance of hairs and lobbed borders [30]. It is well known that the glandular trichomes on the leaves store essential oil. The shrub produces tiny light pink and white blooms [31].

Numerous scientific organizations have actively investigated the promising benefits of *P. cablin* over the years. Numerous types of terpenoids, phytosterols, flavonoids, organic acids, lignins, glycosides, alcohols, and aldehydes are among the many phytochemicals present in *P. cablin*. Originally from Southeast Asia, *P. cablin* Benth. (patchouli) is a significant fragrant herb that has now been extensively grown throughout many tropical and also subtropical areas, including China, the Philippines, Indonesia, and Thailand [32].

The extracted oil, also known as patchouli oil (PO), is the primary focus of analysis and research. Numerous chemicals have been discovered in PO, including PA, trans-caryophyllene, guaiene, caryophyllene, pogostone, elemene, and patchoulene [33]. Sesquiterpenes make up a significant component of PO, while PA has the largest amount of all of the constituents [34]. By using gas chromatography (GC), mass spectrometry (MS), nuclear magnetic resonance (NMR), and other analytical techniques, patchouli’s entire herb, stems, and leaves can be used to isolate PA, a tricyclic sesquiterpene [35]. This is frequently noted as one of the measures to determine the grade of PO and has many bioactivities. Additionally, major sesquiterpenes in PO include patchoulene and patchoulene epoxide. Numerous studies have reported on the physicochemical characteristics and biological actions of these two compounds. Pogostemon is also widely available, and patchouli is another component of patchouli oil that has powerful insecticidal and antibacterial properties [36]. Patchouli contains a number of non-volatile chemicals with substantial biological effect in addition to the volatile oil [37].

In patchouli, more than 50 nonvolatile compounds have been discovered so far, and various analytical techniques have been used to establish their chemical structures. According to their chemical structures, these substances can be loosely classified as terpenoids, glycosides, organic acids, flavonoids, aldehydes, and lignin. Numerous studies have reported on a number of prominent and significant substances, including pachypodol, retusine, ombuin stigmasterol, apigenin, sitosterol, tilianin, isocrenatoside, 3-OMethylcrenatoside, dibutyl phthalate, and tschimganical A [38]. Because of its numerous biological activities, pachypodol has garnered the most interest among them [39]. Pachypodol (5,4′-dihydroxy-3,7,3′-trimethoxyflavone) is extracted from *Croton ciliatoglanduliferus* and *C. floribunda* using mass chromatography, and spectroscopy is used to confirm the structure [40]. Natural polyphenolic chemicals called flavonoids are obtained from plants, and scientists are very interested in their biological activities. Numerous studies have demonstrated the biological benefits of flavonoids, including their anti-inflammatory, anti-fungal, anti-microbial, and antioxidant capabilities [41].

## 3. Pharmacological Activities

**Figure 1:**
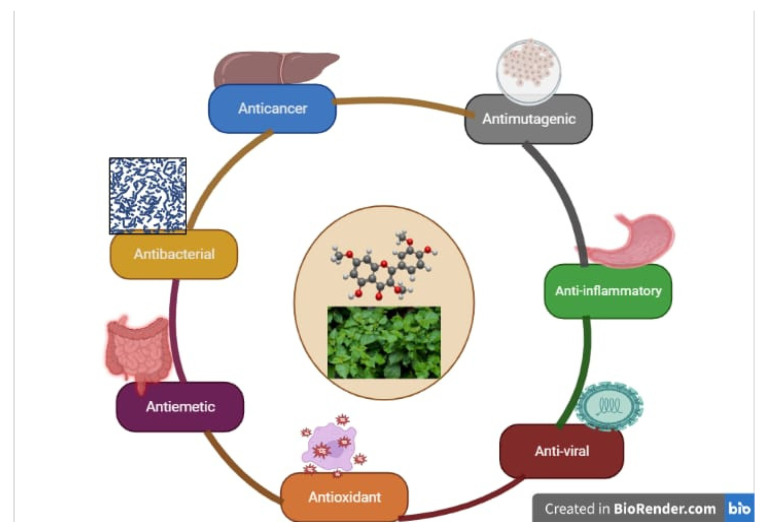
Biological activities of pachypodol.

Numerous experimental studies have examined the pharmacological effects of pachypodol, as represented in Figure 1. For example, 3,7,3′-trimethyl ether (or 4′,5-Dihydroxy-3,3′,7-trimethoxyflavone), belonging to the class of flavonoids, has shown anti-mutagenic activity [18], anti-emetic activity [42], inhibition of water-splitting enzyme [43], cytotoxic activity [44], anti-fungal activity [45], and anti-cancer activity [40]. Kim et al., in 2019 [46], stated that this molecule is highly intriguing due to its defensive properties, which have prompted computer studies on its capacity for radical scavenging. Regarding the molecular structure of pachypodol, the quercetin nucleus has methoxy groups at C-3 by replacing OH- groups, while OH- group is present at the third and seventh carbon (C-3, C-7, and) positions. Pachypodol is a key non-volatile component of *P. cablin*; however, little is known about its pharmacological effects (Table 1).

### 3.1. Antimicrobial Activity

One of the studies, performed by Chuwen et al. in 2013 [58], showed that *P. cablin* leaf extract in ethyl acetate (PCLE) and *P. cablin* leaf extract in hexane (PCLH) total lipid profiling revealed separate bands that suggested the existence of chemicals with different polarities. *Staphylococcus aureus* 6538 (Gram-positive) and *Klebsiella pneumoniae* ATCC 27736 (Gram-negative) bacterial cultures were cultivated in an aseptic environment on nutrient agar using the streak plate technique to isolate discrete colonies of each. Using resazurin as an indicator, PLHE and PLCE were examined for antimicrobial activity towards Gram-negative bacteria and Gram-positive bacteria.

Punithavathy, in 2012 [59], studied the reagent resazurin and used it to count the number of live bacterial cells by the use of tetrazolium compounds. The SA and KP bacteria’s growth was strongly suppressed by both PCL extracts in a concentration-dependent manner according to the resazurin assay. However, compared to PCLH, PCLE had much greater action against both types of bacteria, that is Gram-positive and Gram-negative. Purple resazurin is transformed into the brilliant pink resorufin product by living bacteria. KP for Gram-negative was measured at 0.03 mg/mL [58].

In contrast to PCLH, *P. cablin* leaf extract in ethyl acetate had much greater activity against both types of bacteria (Gram-positive and Gram-negative) [60]. The phytochemicals found in PC leaves that may have antibacterial action should be identified. PCLE was chosen for more research tests. A phytochemical analysis revealed that the PCLE included coumarins, steroids, tannins, flavonoids, glycosides, and carbohydrates. This is consistent with past literature studies where patchouli was used to isolate and identify terpenoids, phytosterols, flavonoids, lignins, alcohols, alkaloids, organic acids, glycosides, and aldehydes [61]. Krithika et al., 2022 [47] showed that numerous phytochemicals in PCLE were also discovered by TLC profiling of the extracts. Therefore, column separation was used to further isolate the phytomolecules contained in PCLE. Fractions 33 to 36 of the pooled fractions showed the existence of a single chemical.

Pachypodol was determined to be the drug following analysis using 1H and 13C NMR spectroscopy. Our data matched the prior reports in which observed NMR spectrum values were compared to published reports [47]. *P. cablin* leaf ethyl acetate extract showed noticeably more anti-microbial efficacy than its hexane extract. Ladan et al., 2014 [62] found that the ethyl acetate extract contained steroids, flavonoids, glycosides, terpenoids, tannins, and carbohydrates, according to phytochemical analysis. Additionally, pachypodol (a flavone derivative and bioactive compound) was found after chromatographic separation and NMR analysis. The research suggests that the EtOAc leaf extract, which contains pachypodol, may have antibacterial properties [47].

### 3.2. Anti-Mutagenic Activity

Pachypodol’s antimutagenic properties were easily identified. Pachypodol was measured in the umu test using *Salmonella typhimurium* TA1535/pSK1002 and mutation-causing chemicals, including furylfuramide and Trp-P-1. It is suggested that the development of suppression effects in the umu test was likely caused by the single methoxy group (-OCH3) at the 4′-position of the aromatic ring [63].

Pachypodol demonstrated strong SOS induction inhibition in the litter mutagen at a lower dose than that of furylfuramide. At 0.06 mol/mL, pachypodol reduced >80% of Trp-SOS-inducing P-1 activity [64]. Compared to furylfuramide, pachypodol had stronger inhibitory impacts on the Trp-P-1-induced SOS response.

The Ames test with *S. typhimurium* TA100 also showed that pachypodol has anti-mutagenic properties against furylfuramide, activated Trp-P-1, and Trp-P-1 [57]. Pachypodol had less inhibitory effect on activated Trp-P-1 than Trp-P-1 did. Against activated Trp-P-1, it exhibits very modest inhibitory effects.

Pachypodol was tested for its capacity to block Trp-metabolic P-1’s activation by S9. Pachypodol failed to prevent activated Trp-P-1 from being induced into SOS. These findings pointed to two potential outcomes: the -OH, or sometimes -OCH_3_, group at the third position increases the repressive impact against Trp-P-1; the reduction of Trp-ability P-1’s ability to activate S9’s metabolism prevents it from causing SOS. This discovery raised the idea that S9’s inhibition of metabolic activation was what produced Trp-inhibition P-1’s inhibition of SOS-inducing activity, which was brought on by pachypodol [18].

### 3.3. Anti-Oxidant Activity

Free radicals, also called ROS, are produced normally by cellular metabolism from oxygen [65]. The main ROS and NOS are nitric oxide (NO), hydrogen peroxide (H_2_O_2_), hydroxyl radical (OH), and superoxide anion (O^2−^) [66]. OS happens when the body’s antioxidant defense mechanism and ROS levels are incompatible. Jaiswal et al. (2004) [67] state that antioxidant defense mechanisms, including SOD, CAT, GSR, and GPx, serve as the body’s fundamental source of defense and shield biomolecules (lipids, proteins, and DNA) from OS by reducing excessive ROS production. An essential free-radical-scavenging enzyme called SOD turns O_2_ into H_2_O_2_, which is then changed into water by CAT [68]. By reducing glutathione (GSH) into glutathione disulfide (GSSG), GPx aids in the breakdown of H_2_O_2_. GSH, on the other hand, serves in these processes as an electron donor. GSR maintains the concentration of GSH, which is essential for the generation of sperm [69].

Perfluorooctane sulfonate (PFOS) intoxication is reported to result in increased degrees of abnormalities in the head, mid-piece, and tail of sperm [70], as well as a decrease in feasibility, motion, epididymal sperm [71], and HOS coiled-tail sperm count. Agarwal et al., 2020 [72] stated that the OS significantly contributes to testicular dysfunction. The distorted integrity of sperm may be brought on by an imbalance between antioxidants and oxidants, which leads to membrane damage because of the elevated levels of polyunsaturated fatty acids (PUFAs) in sperm. Ijaz et al., 2021 [73] performed experiments and evaluated the possible therapeutic advantages of pachypodol in the case of perfluorooctane sulfonate (PFOS)-caused injury in the testicles of adult male rats. Rats (*n* = 48) were randomly assigned to a group (control, PFOS, PFOS + pachypodol, or pachypodol alone), each of which had 12 rats. In this experiment, PFOS at a dose of 20 mg/kg and pachypodol at a dose of 10 mg/kg were both administered orally. The powerful ROS-scavenging activity of pachypodol allowed for the effective restoration of all spermatogenic defects [73].

Wan et al., 2020 [74] have studied and found that the vitality, motility, number of HOS (hypo-osmotic swelling) coiled-tail sperms, and epididymal sperm count all significantly decreased in response to PFOS (perfluorooctane sulfonic acid) poisoning, but a higher amount of abnormalities were seen in the head, mid-piece, and tail of sperms. Additionally, it decreased follicle-stimulating hormone, plasma testosterone, and luteinizing hormone. Additionally, exposure to PFOS caused testicular histological damage [74]. The pachypodol therapy, however, effectively reversed all of the testicular deficits shown. In conclusion, findings showed that a new phytochemical called pachypodol has promising free-radical scavenging properties against the testicular dysfunctions caused by PFOS [71].

Another study, by Frish et al., 2009 [75], explained that anti-oxidant activity is connected to the compound’s protective function and free-radical-scavenging ability. The basis set 6-311G (d,p) and the hybrid meta-exchange correlation functional (M06-2X) and hybrid exchange correlation functional (B3LYP) approaches were used to optimize neutral compounds, radicals, anions, and cations. These methods were implemented in the Gaussian 09 program. The Gaussian 09W computational tool used DFT (density functional theory) to investigate the antioxidant-related properties of pachypodol [75]. The mechanisms of three radical-scavengers SPLET, HAT, and SET-PT were more preferable. According to research on anti-oxidant activity, there are three basic ways that phenolic anti-oxidants (ArOH) neutralize free radicals [76]. With direct hydrogen atom transfer (HAT) to the radical (R), the deprotonation occurs before the electron transfer, which happens after the anion is generated according to the SPLET18-20 mechanism. SET-PT involves the reverse process, in which phenolic antioxidants create a radical cation by electron transfer that quickly deprotonates to create a phenoxyl radical [77].

According to one hypothesis, the heterolytic BDE in which the 4′-OH has been shown to have the least magnitude radical is resolved by the SPLET mechanism created by the addition of PA and ETE. Additionally, the radical 4′-OH is known to have the lowest PA magnitude [78]. Since the 4′-OH radical in pachypodol transfers protons more readily than the -OH radical, it is determined after examining the SET-PT mechanisms of both quantum approaches that to determine the ideal location for electron and proton transfer from chemical, IP (ionization potential) and PDE (partial differential energy) are essential components [79].

The electron transfer is easier when IP is smaller, which accounts for the initial phase of the SET-PT antioxidant mechanism. It should be observed that both theories demand less energy for the release of the H-atom (BDE) than for a single electron transfer (IP). Extended delocalization and conjugation of electrons are to blame for this. When using the information from the second stage of SET-PT to determine the likely site for deprotonation, it is crucial to find that the PDE of 4′-OH is at a minimum. It has been found that that 4′-OH radical is the preferable site as demonstrated by HAT, SPLET, and SET-PT [78].

Furthermore, Bryan et al., 2013 [52] studied the ability of GSH (a substantial non-protein antioxidant endogenous compound) to reinforce the intracellular immune system that has been explained through the stimulation of antioxidant Nrf2-modulated enzymes and a large observed increase in GSH levels. Because pachypodol treatment improves the Nrf2-dependent endogenous antioxidant defense system, which has previously been reported to be significantly reduced by t-BHP treatment and causes intracellular depletion of GSH, which causes oxidative damage, pachypodol treatment may have protective effects towards oxidative stress induced by t-BHP. Meanwhile, subsequent hepatocyte cell death is also protected by the use of pachypodol [52].

**Figure 2:**
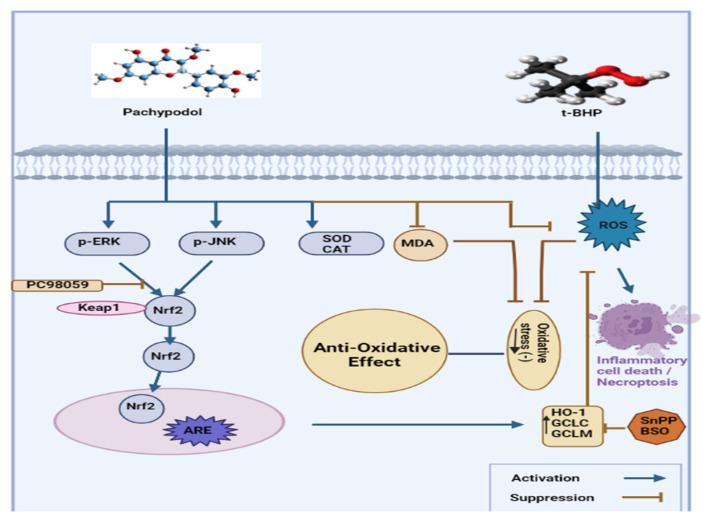
Anti-oxidative pathway activation by pachypodol.

It has been established that a number of kinases, such as ERK, PI3K/Akt, AMPK, PKC, PERK, GSK3, and Fyn kinase, are involved in a number of processes that control the transcriptional activation of Nrf2 [80]. The results shows that pachypodol completely inhibited the increase in nuclear Nrf2 expression, enzyme activity by ARE-luciferase, and mRNA expression by GCLC and GCLM, indicating that ERK is the main cause of pachypodol-induced Nrf2/ARE activation (Figure 2) [46]. Further evidence for this crucial function of ERK activation came from pachypodol-stimulated ERK phosphorylation. The Keap1-Nrf2 complex is reported to be broken down by ERK, which then facilitates Nrf2’s nuclear translocation [81].

The significance of the ERK signaling pathway to regulate Nrf2 activation has also been shown in numerous additional studies, as described in Figure 2. By examining the pharmacological effects of different phytochemicals such as quercetin, epigallocatechin gallate, sulforaphane, and nectandrin, B. Gu et al., 2012 [82] determined that the liver is exposed to ROS constantly because it is a crucial place for the metabolism of xenobiotics and endogenous substances. Pachypodol, produced from *P. cablin* Bentham, is a methoxyflavonoid and can thereby guard against oxidative damage to hepatocytes. This defense might be strengthened by activating Nrf2 in an ERK-dependent manner, which would increase endogenous antioxidant defense [81].

### 3.4. Anti-Viral Activity

Sandoval et al., 1997 [83] stated that the phosphatidylinositol 4-kinase III beta (PI4KB) has just come to be recognized as an important target of anti-picornavirus medications. In the current work, it was discovered that the anti-picornavirus drug pachypodol directly targets PI4KB. Using a kit from ADP-Glo Lipid Kinase Systems, the inhibitory effects of anti-PV drugs on in vitro PI4KB activity were assessed (Promega). In an experiment, 10% FCS was added to Dulbecco’s Modified Eagle Medium, while a human rhabdomyosarcoma cell line (RD) and human embryonic kidney cells (HEK293) were grown in monolayers. The feasibility of the treated compound cells and the calculation of the CC50, as previously described, were used to assess the cytotoxicity of the substance [84]. According to reports, pachypodol directly targets PI4KB rather than PI3Ks in order to prevent BFA-induced Golgi disassembly, which was first observed by Sandoval et al. (1997) [83]. Inhibitors of PI4KB/OSBP treatment resulted in a decrease in PI4P in the Golgi apparatus, indicating that PI4P in the Golgi apparatus, as opposed to PI4KB activity, is crucial for BFA-induced Golgi disassembly [49].

### 3.5. Anticancer Activity

Due to their anti-proliferative and pro-apoptotic qualities, flavonoids extracted from *P. cablin* (such as pachypodol 13), natural antioxidants, and other phytochemicals have lately been recommended as anti-cancer complementary treatment therapy [85,86]. Therefore, as natural herbal remedies have numerous benefits, the ongoing quest for anticancer agents or molecules from plants has played a crucial role in discovering possible ways to ensure the safety of and to lessen the unwanted effects caused by chemotherapy [87]. Studies on plants such as *P. cablin* (and another plant named mistletoe, an obligate semiparasite plant) have found that its extracts, which contain alkaloids, viscotoxins, lectins, and polysaccharides, have a variety of biological potentials, including the ability to suppress cancer. The main anti-cancerous components in mistletoe include pachypodol, betulinic acid, and morpholic acid [88]. These plants have gained popularity as a potential complementary treatment for malignancies of the mouth, throat, lungs, colon, breast, and pancreas, and for endometrial and colorectal cancer [89]. The extract of the plant improves immunity, postpones the onset and progression of malignancies, eradicates malignant tumors, stabilizes DNA, lessens chemotherapy side effects, and increases the resiliency of cancer patients and survivors. Iscador, eurixor, helixor, lektinol, isorel, iscucin, abnoba-viscum, and recombinant lectin ML-1 are only a few of the proprietary medications presently available for purchase [88].

*Miliusa balansae* is a shrub of the family Annonaceae. It is used in ancient Chinese remedies for gastropathy and glomerulonephropathy [90]. Recently, some flavanones, dihydrochalcones, and flavones were extracted from *Miliusa balansae* [91,92]. Compound **1** was derived as a yellow amorphous solid. Its IR spectrum showed the presence of hydroxyl groups and carbonyl group. The molecular formula of 1 was determined to be C_25_H_22_O_8_. From spectral data, we can conclude that compound 1 is a C8-o-benzyl derivative of pachypodol, which has also been isolated from this plant [93]. This conclusion was further supported by comparison of a part of the 1 H and 13C NMR data (ppm) of 1 and pachypodol (4). The new compound 1 has been named miliufavol. Besides compound **1**, ombuine (2), chrysosplennol B (3), pachypodol (4), and chrysosplenol C (5) were derived from this plant. The cytotoxicity of compounds **2**–**5** against cancers, such as human epidemoid carcinoma, hepatoma-G2, and rhabdosarcoma was investigated. These assays are established on the method of Likhiwitayawuid et al (1993) [94] and Skehan et al (1990) [95]. According to the findings, all four compounds were effective against the three cell lines that were put to the test. Pachypodol (4), among them, exhibits potent effects on two cell lines (KB: 0.7 mg/mL, Hep-G2: 0.55 mg/mL). This made it intriguing to conduct additional research on the cytotoxic effects of pachypodol [44]. Pachypodol, a flavone found in *P. cablin* and Calycopteris floribunda leaves, was found in a study to suppress the growth of the CaCo-2 colon cancer cell line in vitro. Using the brine shrimp lethality assay and Promega’s Cell Titer 96 non-radioactive cell proliferation assay, the toxicity of the isolated flavonoid compound pachypodol was evaluated in the cancer coli-2 (CaCo-2) colon cancer cell line. The chemical has median lethal dose (LD50) values of 435.8 M for general toxicity and 185.6 M for cytotoxicity. As a result, the substance has a mild cytotoxic effect on the CaCo-2 colon cancer cell lines [40]. Phytochemical studies found pachypodol and other biflavonoids of the other kinds, such as calycopterones and calyflorenone, in plants such as *P. cablin* [77,96]. It has been discovered that several of these flavonoids and bioflavonoids have anti-tumor effects ([97,98,99]. This flavonoid (1) may not be a good option for the creation of therapeutically important and commercially applicable anti-cancer medicines in its current form, however, because it exhibited cytotoxicity toward the CaCo-2 cells at moderate levels. However, it is possible to create molecules with increased activity by taking advantage of the structural characteristics this flavonoid possesses [40]. To clarify the links between structure and cytotoxic activity, as well as to identify the pharmacophore(s) present in these molecules, it is possible to test for cytotoxic activity in different naturally occurring flavonoids that are structurally related to compound 1. Although pachypodol (1)’s anti-colon-cancer action has not been described in the past, this flavonoid is considered to have a number of other biological functions [100].

### 3.6. Apoptosis Induction

**Figure 3:**
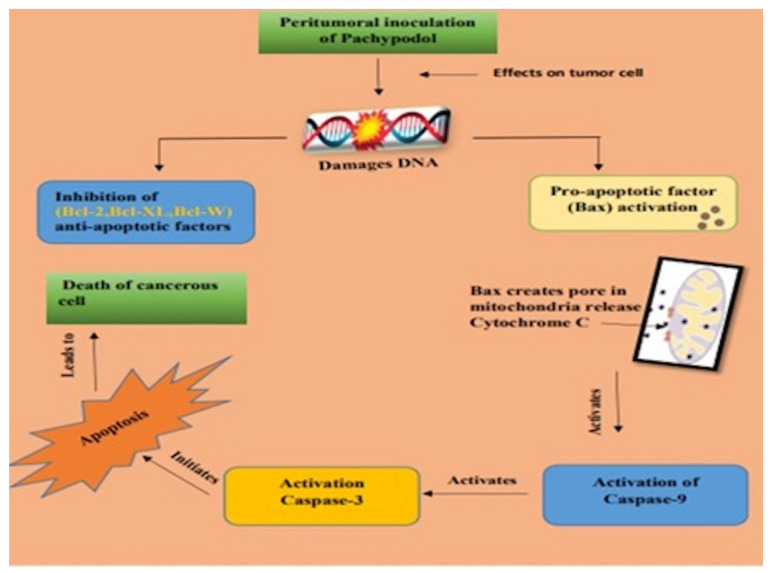
Apoptosis pathway activation by pachypodol.

Research using cell culture and animal specimens suggests that pachypodol’s cytotoxic and anti-cancer effects may be regulated by a variety of processes, including the induction of apoptosis and necrosis (Figure 3), activation of the specific or general immune system, inhibition of cell cycle progression, and beta-endorphin release into the blood [101].

Increased cancer observation, anti-angiogenic anticancer, and apoptotic activities have been seen in a variety of *P. cablin* species. Different suppressive effects have been seen against pancreatic, lung, oral, and colon malignancies [46]. The administration of certain commercial products is done in conjunction with supplementary oncotherapy. The most popular way to administer mistletoe extract is through intrathecal injections. The extracts are injected into the tumor, vein, pleural cavity, or skin. Through a cohort analysis, Friedel et al. (2009) [102] assessed the effectiveness of the therapy with the mistletoe extract iscador in patients with nonmetastatic colorectal cancer. Human leukemia K562, human plasmacytoma RPMI-8226, and murine lymphocytic leukaemia L1210 cells in culture all experienced decreased survival and apoptosis as a result of the treatment. The p38 MAPK, JNK-1/2, and caspase-9 were activated as part of the intrinsic apoptosis process, accompanied by the suppression of ERK-1/2 and PKB phosphorylation and downregulation of Mcl-1 [46]. Antimutagenic protection against mutagens, for which pachypodol has been quite effective, is of significant importance in cancer prevention [103].

### 3.7. Anti-Inflammatory Activity

The anti-inflammatory potential of pachypodol was studied as one of the extracts from a plant known as Patchouli, also known as “Guanghuoxiang”, which is scientifically known as *P. cablin Benth* and is a member of the Lamiaceae family. Among the bioactive substances found in patchouli are glycosides, terpenoids, favonoids, alcohols, phytosterols organic acids, lignins, pyrone, and aldehydes. One of these compounds, pachypodol, is also quite important. It was discovered to have various pharmacological properties (e.g., an anti-inflammatory impact) [104].

Pachypodol’s anti-inflammatory properties were also reported when extracted from the leaves of the plant *Aglaia andamanica*. Nitric oxide (NO) is an inflammation mediator that induces swelling in several organs. As a regulatory substance with homeostatic functions, NO functions as a host defense [105]. However, overproduction of this free radical is harmful because it can attach to superoxide radicals and interfere with the regular functioning of cells [106]. Then, the anti-inflammatory properties of the Aglaia andamanica chemical extracts, including pachypodol, yangambin, 24-epi-piscidinol (A) pyramidaglain (A) and pyramidaglain (B), were examined using RBL2H3 and RAW264.7 cells; pachypodol had strong anti-inflammatory activity, with an IC50 of 34.5 M and 24.0 M, respectively. Pachypodol showed cytotoxicity at high concentrations (100 M) (25–30%) when compared to the positive controls. Pachypodol showed stronger action against NO generation; nevertheless, it was less efficacious than CAPE, an NF-kB inhibitor [46]. The current study concludes by demonstrating the significant anti-allergic and anti-inflammatory properties of *A. andamanica* [8].

Pogostemon patchouli methanol extracts, such as ombuine and pachypodol, are phenolic chemicals with antibacterial, antifungal, and anti-inflammatory properties. These were assessed using the formalin test, acetic-acid-induced writhing reaction, and carrageenan-induced mice paw edema. The outcomes of the experiments suggested two potential mechanisms for the anti-inflammatory effect. One involves limiting the quantity of arachidonic acid (AA) converted to prostaglandins (PGs) by inhibiting the expression of various inflammatory mediators, such as TNF (tumor necrosis factor), IL (interleukin)-1, iNOS (inducible nitric oxide synthase), and COX (cyclooxygenase)-2, which results in PGE2 (prostaglandin E2). The second method involves getting rid of free radicals by increasing the activity of antioxidant enzymes, including glutathione peroxidase, superoxide dismutase, and GPx [56].

### 3.8. Antiemetic Activity

Pogostemon patchouli (PP), a traditional Chinese medicine, has antiemetic properties and is typically used to treat dyspepsia, vomiting, diarrhea, and poor appetite. In a research study, chicks were given copper sulphate (CuSO_4_) to induce vomiting [42]. An n-hexane extract of PP (Pogostemon patchouli), which contains pachypodol, was tested for antiemetic activity, and it demonstrated the highest antiemetic activity (58.6%), as compared to methanol and water extract. Patchouli alcohol (PA), which is abundant in PP and constitutes more than 0.1 percent, may play a significant role in the antiemetic activity. It is well recognized that inward Ca^2+^ influxes through cell membranes can lead to excessive smooth muscle stimulation, and that too much contraction of the muscles of the digestive system can result in nausea, vomiting, and diarrhea [107]. These findings suggest that pachypodol’s antiemetic properties may derive from its ability to reduce excessive muscular contraction in the digestive system by preventing inward Ca^2+^ influx via the cell membranes, as is the case with patchouli alcohol [57].

In a study using a chick model of copper-sulfate-induced emesis, the antiemetic efficacy of *Peltophorum roxburghii* L. leaves extracted in methanol was evaluated. The plant contains a variety of substances with antiemetic properties, including pachypodol, bergenin, galangin, kaempferidec, and reustin. Through antagonistic 5-HT3, 5-HT4, or NK1 receptor action, pachypodol has demonstrated antiemetic efficacy. Due to their antioxidant properties, quercetin and reustin demonstrated a similar impact. While the conventional medicine chlorpromazine showed 46.62–32.70% inhibition, bergenin (25 mg/Kg p.o.) revealed 20.20 retches with 70.84% inhibition. Both bergenin and chlorpromazine had a notable (*p* < 0.05) impact. As a result, substances derived from Peltophorum roxburghii leaves exhibit notable antiemetic action [56].

## 4. Conclusions

Pachypodol is a natural flavonoid compound that has been identified in several plant species, most importantly Pogoston patchouli benth and others including Mistletoe, *Miliusa balansae*, *Pteris ensiformis*, and *Eriocaulon sieboldianum*. It has been found to have various biological activities, such as antioxidant, anti-inflammatory, and antitumor properties. Pachypodol has also been reported to have potential therapeutic effects on certain diseases, such as Alzheimer’s, diabetes, and colon cancer, as well as spermatogenic defects, through its ability to modulate various signaling pathways in the body. In addition, it has shown a moderate level of biological activities. Pachypodol, with its additional significant chemicals, also contributes to the biological activity of patchouli. Nonetheless, additional investigation of pachypodol’s chemical structure and derivatives is necessary to determine its significance as a single major compound found in *P. cablin* for its biological activities. In addition, further research is required to completely understand its role in biological pathways and its potential applications.

## Figures and Tables

**Table 1:** Various pharmacological activities of pachypodol determined using different assays.

Activities	Tests	Target	Results	References
Antimicrobial activity	Resazurin assay	*Staphylococcus aureus* 6538 and *Klebsiella pneumonia* ATCC 27736	Growth inhibited	[47]
	Resazurin assay	*Staphylococcus aureus* (MTCC 737), *Bacillus subtilis* (NK-1), *Salmonella enterica* (ATCC 14028), and *Escherichia coli* (MTCC 1302).	Low bacteriostatic effect against bacteria strains, except for *B. subtilis,* against which it showed a moderate inhibition effect	[48]
Antiviral activity	ADP-Glo Lipid Kinase Systems kit	Phosphatidylinositol 4-kinase III beta (PI4KB)	Interferes with brefeldin A (BFA)-Golgi disassembly, BFA targets viral RNA synthesis	[49]
Anti-mutagenic	Ames test	*S. typhimurium* TA100	Suppress SOS-inducing activity	[18]
Antioxidant	Terminal deoxynucleotidyl transferase nick-end labelling (TUNEL) assay	Rats	Cytokine levels and Oxidative stress reduced	[50]
	Cell-based assays	HepG2 cells	Pachypodol protected HepG2 cells from cell death caused by oxidative stress and reduced ROS production	[46]
	Superoxide anion radical-scavenging method	Leaves of *C. floribunda* extracts	High antioxidant activity	[51]
	HAT, SPLET, AND SET-PT	Rats	ERK activation, Nrf2-regulated enzyme activation, increase in GSH levels	[52]
Anti-cancerous	MTT assays	Human ovarian cancer cells (A2780)	Inhibition of cell growth	[53]
	Brine shrimp lethality assay and Promega’s Cell Titer 96 non-radioactive cell proliferation assay	Cancer Coli-2 (CaCo-2) colon cancer cell line	Showed high toxicity against Cancer Coli-2 (CaCo-2)	[51]
	MTT assay	Chinese mistletoe MCF7 cell line	Positive cytotoxic effect	[54]
	Likhiwitayawuid method, ELISA	Human cell lines; human epidemoid carcinoma (Kb), Hep-G2 (Hepatoma-G2), RD (Rhabdosarcoma)	Showed strong cytotoxicity against cancer cell lines	[44]
Anti-inflammatory	MTT assays	RBL2H3 and RAW264.7 cells of *Aglaia andamanica* leaves	Pachypodol exhibited cytotoxicity (25–30%) at 100 mM	[8]
	Nitric oxide (NO) production assay	Leaves of *Eupomatia laurina* tree	High levels of cell growth inhibition.	[55]
	Formalin test	TNF, IL-1, COX-2, PGE2, SOD and glutathione peroxidase	Inhibited expression of inflammatory mediators	[56]
Anti-emetic	New assay method	Young 4-day-old male chicks	5-HT3, 5-HT4, or NK1 receptor antagonism	[56]
	Screening	Young chicks	Anti-emetic properties are demonstrated at doses of 10–50 mg/kg	[15]
	Antagonistic 5-HT3, 5-HT4, or NK-1 receptor action test	Digestive organ muscles	Inward Ca^2^^+^ efflux through cell membranes	[57]

## Data Availability

No new data were created or analyzed in this study. Data sharing is not applicable to this article.
